# DNA demethylation in the PTEN gene promoter induced by 5-azacytidine activates PTEN expression in the MG-63 human osteosarcoma cell line

**DOI:** 10.3892/etm.2014.1571

**Published:** 2014-02-21

**Authors:** DEYE SONG, JIANGDONG NI, HONGMING XIE, MULIANG DING, JUN WANG

**Affiliations:** Department of Orthopaedics, The Second Xiangya Hospital, Central South University, Changsha, Hunan 410011, P.R. China

**Keywords:** DNA demethylation, PTEN gene, PTEN expression, 5-azacytidine, MG-63 human osteosarcoma cell line

## Abstract

This study used the MG-63 osteosarcoma cell line to investigate the demethylation of the phosphate and tension homolog (PTEN) gene promoter and the change in PTEN gene expression levels, which are caused by the methylation inhibitor 5-azacytidine (5-Zac), and the association between the two. Different concentrations of 5-Zac (0, 5 and 10 μmol/l) were added into the MG-63 cell culture medium and the cells were cultured for 72 h. The following techniques were performed on the cells: Western blot analysis to detect the PTEN protein; reverse transcription-polymerase chain reaction (PCR) to detect the mRNA transcription levels of the PTEN gene; flow cytometry to detect the cell apoptotic rate; and sodium bisulfate to deal with the DNA of each group. The genes of the PTEN promoter and the transcription factors specificity protein 1 (Sp1) and Myc were PCR amplified and transformed into *Escherichia coli*, then a number of clones were selected for sequencing and the methylation status of the amplified PTEN promoter fragment was detected. Following culture of the MG-63 cells with 5-Zac at concentrations of 0, 5 and 10 μmol/l for 72 h, the expression levels of PTEN protein in each group were gradually increased, presenting a concentration-dependent effect: Group 0 μmol/l compared with groups 5 and 10 μmol/l, P<0.05; and group 5 μmol/l compared with group 10 μmol/l, P=0.007. The mRNA expression levels of the *PTEN* gene significantly increased. The apoptotic rates of groups 0, 5 and 10 μmol/l were 0.69±0.42, 2.50±0.30 and 6.59±0.62%, and significant differences (P<0.01) were observed between every two groups. The bisulfate DNA sequencing results of three groups showed that, following the treatment with 5-Zac, the binding of the CG site to transcription factors was affected by demethylation. The average rate of demethylation indicated a statistical difference among the three groups. In conclusion, the methylation inhibitor 5-Zac leads to a significant increase in the expression levels of the tumor suppressor gene PTEN in the MG-63 osteosarcoma cell line *in vitro*. The expression levels of mRNA and the cellular apoptotic rate were also increased. The elevated activation and expression levels of the PTEN gene may be associated with the low methylation levels of the CG site that binds to the transcription factors Sp1 and Myc in the PTEN gene promoter, and they promote the combination of the transcription factors and the gene promoter.

## Introduction

Osteosarcoma is the most common type of primary bone tumor and causes serious harm to the health of adolescents. Osteosarcoma is highly invasive and is transferred by the blood in the early stage, and progresses rapidly. It mainly occurs in actively growing long bone metaphysis. This type of tumor has a high degree of malignancy, recurrence and metastasis and the prognosis is poor. The incidence was reported in 2009 as ~4 million individuals per year (1). Osteosarcoma cells have strong invasive ability, quick hematogenous metastasis in early stage, rapid progression, and the five-year survival rate was only 60% in 2008 (2). Although treatment of osteosarcoma has been on the increase, the five-year survival rate remains low, and the recurrence rate is high (3). Therefore, investigation of the pathogenesis of osteosarcoma and attempts to identify a novel approach to reduce the tumor recurrence rate and improve the survival rate of the patients is of significant importance in the clinic.

The development of osteosarcoma is complex, and the molecular mechanism is not clear yet. Numerous studies have shown that there are abnormal expression levels of the phosphate and tension homolog (PTEN) gene in human osteosarcoma cells or tissues. The *PTEN* gene deleted on chromosome 10, also known as mutated in multiple advanced cancer 1 and TGF-β-regulated and epithelial cell-enriched phosphatase, is located on chromosome 10q23.3. The gene consists of nine exons, encodes a protein which is composed of 403 amino acids and has phosphatase enzyme activity (4,5).

The *PTEN* gene was first identified in 1997 (6), and it is considered an important tumor suppressor gene together with p53 and Rb. It is also the first tumor suppressor gene with phosphatase activity to be observed thus far.

The PTEN protein inhibits tumor occurrence and development mainly through the following three pathways: i) Inositol triphosphate kinase [phosphoinositide 3-kinase (PI3K)/AKT]pathway. The protein encoded by PTEN has lipid phosphatase activity, thus it competes with PI3K and causes dephosphorylation of phosphatidylinositol (3,4,5)-triphosphate (PIP3), and this prevents the growth factors’ signal transduction pathway regulated by PI3K. The reduced PIP3 levels arrest the cell in G1 phase, thereby inducing apoptosis of the tumor cells. ii) Mitogen-activated protein kinase (MAPK) pathway. PTEN inhibits the upstream extracellular signal-regulated kinase (ERK) of MAPK, the activation of Ras and the phosphorylation of Shc. The PTEN gene also inhibits the phosphorylation of MAPK kinase and blocks the cell in G1 phase, thus inhibiting tumor growth (6–8). iii) Focal adhesion kinase (FAK) pathway. FAK is an important factor in the integrin-mediated signal transduction pathway. Activated FAK activates several associated kinases and signaling molecules that promote cell invasion and metastasis. PTEN inhibits the activation of FAK by causing its dephosphorylation, thus inhibiting the invasion and metastasis of tumor cells.

The abnormal expression levels of the PTEN gene play an important role in tumor occurrence and development (9). A study concerning the expression levels of PTEN in osteosarcoma tissues has demonstrated that there is a significant reduction in the levels of PTEN protein expression in osteosarcoma tissue. Through enhancement of the phosphorylation levels of Akt, PTEN is inhibited and thus promotes the proliferation of osteosarcoma cells (10). The reason for the expression levels of the PTEN gene being abnormally low in osteosarcoma tissues remains unclear.

Studies have confirmed that hypermethylation of tumor suppressor genes is closely associated with the occurrence of tumors and the methylation levels of the CpG islands in eukaryotic DNA are closely associated with cell canceration (11–12). If there is an unmethylated CpG island in a tumor suppressor gene, this tumor suppressor gene easily becomes the attack target of DNA methyltransferases (DNMTs). In cancer cells, the activation levels of DNMTs are increased, tumor suppressor genes show hypermethylation status of the CpG islands and cause transcriptional inactivation. A number of studies have confirmed that the abnormal methylation of the PTEN gene promoter leads to abnormal gene expression levels (13–15) and the methylation of the promoter enhancer region CpG islands causes certain transcription factors to be unable to bind to DNA and thus inhibits gene transcription.

Myc and Sp1 are the main transcription factors in the PTEN promoter that regulate the transcription of PTEN (16,17). To the best of our knowledge, it has not been reported whether DNA demethylation influences the expression levels of the PTEN gene in osteosarcoma cells and the methylation degree of the GC site that binds to Myc and Sp1 in the PTEN promoter. To the best of our knowledge, there are few studies concerning the epigenetic changes of PTEN in osteosarcoma, i.e., whether the methylation status of the PTEN gene promoter region affects the expression levels of the PTEN protein, and if it does, the mechanisms by which this happens.

The cultured MG-63 osteosarcoma cell line was used for the present study. The growth inhibition and induced apoptosis caused by different concentrations of 5-Zac added to MG-63 cells were observed, and the changes in the PTEN gene mRNA and the expression levels of the PTEN protein were detected. Bisulfite sequencing was further used to detect the methylation status of the CG site for binding to the transcription factor Myc in the PTEN gene promoter, and the associations between them.

## Materials and methods

### Materials

#### Cell line

The MG-63 osteosarcoma cell line was provided by the Cell Culture Centre of Xiangya School of Medicine, Central South University (Changsha, China).

#### Reagents and instruments

5-Zac, formula C_8_H_12_N_4_O_5_ and relative molecular weight 244.205, was purchased from Sigma-Aldrich (St. Louis, MO, USA). RPMI-1640 medium and fetal bovine serum Australia origin were purchased from Gibco (Carlsbad, CA, USA). Rabbit anti-human polyclonal and anti-PTEN antibodies were purchased from Millipore (Billerica, MA, USA), and rabbit anti-human anti-β-actin and peroxidase-conjugated goat anti-rabbit anti-IgG were purchased from Santa Cruz Biotechnology, Inc. (Santa Cruz, CA, USA). A DNA extraction kit, Taq enzyme, dNTPs and a reverse transcription kit were purchased from Promega GmbH (Madison, WI, USA). DNA reference standards were purchased from Fermentas (Burlington, Canada). TRIzol was purchased from Invitrogen (Carlsbad, CA, USA) and a sodium bisulfite treatment kit was purchased from Chemicon American Companies.

### Methods

Cell culture. The MG-63 cells were cultured in RPMI-1640 medium with 10% fetal bovine serum, and incubated at 37°C in a humidified atmosphere with 5% CO_2_. After the cells covered the bottom of a 9-cm petri dish, they were subcultured in a 6-cm dish and the medium was changed every 2–3 days. Experimental intervention was exerted when the cells reached 60–70% fusion.

#### Methylation inhibitor 5-Zac processing

There were three treatment groups in the study. 5-Zac with a final concentration of either 0, 5 or 10 μmol/l was added to the medium of the cells, and then the cells were incubated at 37°C in a humidified atmosphere with 5% CO_2_. The culture medium and the same concentration of 5-Zac were changed every 24 h, and after 72 h the treatment the cells were harvested and tested. Each experiment was repeated three times.

#### Flow cytometry to detect the apoptotic rate of the MG-63 cells

Following trypsin digestion, 1×10^6^ MG-63 cells were harvested, washed twice with ice-cold phosphate-buffered saline (PBS), fixed and permeabilized with 70% ethanol at −20°C for 24 h, and washed once with ice-cold PBS. After incubation with propidium iodide (PI) staining buffer at 37°C for 1 h, the cells were washed one more time with ice-cold PBS and DNA content analysis was performed with a FASCalibur Flow Cytometer (Becton, Dickinson and Company). The PI staining buffer contained 1X PBS, 100 μg/μl RNase and 40 μg/ml PI.

#### RNA extraction and reverse transcription-polymerase chain reaction (RT-PCR)

The total RNA was extracted with TRIzol according to the manufacturer’s instructions. Following quantification by UV spectrophotometry, 1 μg of the total RNA was used for reverse transcription reaction synthesis of cDNA with a reverse transcription kit, according to the manufacturer’s instructions. PCR was used to amplify the cDNA. The corresponding primer sequences were as follows: forward, 5′-CCACCCATGGCAAATTCCATG-3′ and reverse, 5′-TCTAGACGGCAGGTCAGGTCCACC-3′ for reference GAPDH; and forward, 5′-TTGAAGACCATAACCCACCA-3′ and reverse, 5′-CACATAGCGCCTCTGACTG-3′ for PTEN.

Quantitative PCR was performed using a Rotor-Gene 3000 Real-Time PCR instrument (Corbett Research, Australia). PTEN and β-actin mRNA were amplified by SYBR-Green real-time PCR using the One Step PrimeScript RT-PCR kit (Takara Biotechnology Co., Ltd., Dalian, China). GAPDH mRNA was used as the internal control. The reactions used the following cycling conditions: 94°C initial denaturation for 3 min, 94°C denaturation for 30 sec, 60°C annealing for 30 sec and 72°C extension for 30 sec for a total of 35 cycles, and a final extension at 72°C for 7 min. Relative PTEN mRNA expression levels normalized to those of β-actin mRNA were calculated using the equation: 2^−ΔΔCt^, where ΔΔCt(relative quantification) = (CT_PTEN_ - CT_β-actin_)_CHB patient_ - (CT_PTEN_ - CT_β-actin_)_Normal control_

#### Western blot analysis of the expression levels of PTEN protein in MG-63 cells

The MG-63 cells treated with different concentrations of 5-Zac were cultured and collected, and the protein samples were extracted by radioimmunoprecipitation assay lysate. The bicinchoninic acid (BCA; Keygen Biotech, Nanjing, China) method, using a microplate reader (Thermo, Waltham, MA, USA) at 570 nm wavelength, was used to detect the total protein concentration. Protein (20 μg) was collected from each group, electrophoresized in 12% SDS-PAGE and a wet electrostatic transfer method was used to transfer the protein to a nitrocellulose membrane. Non-fat milk (5%) was used to block the membrane at room temperature for 1 h, then anti-PTEN (working concentration, 1:500) and internal reference anti-β-actin (1:2,000) were added to the membrane and it was incubated at 4°C overnight. The membrane was washed in PBS three times, each time for 10 min, and peroxidase-labeled anti-IgG was added as the secondary antibody (working concentration, 1:1,000). The membrane was incubated for 1 h at room temperature and washed with PBS three times, each time for 10 min. An ECL chemiluminescence kit (Thermo) was used to develop the membrane. The experiment was repeated three times.

#### DNA extraction and bisulfate sequencing to detect the methylation status of the PTEN gene fragment

The genomic DNA of the cells was extracted using a DNA extraction kit according to the manufacturer’s instructions. Following identification and quantification by UV spectrophotometry, 1 μg DNA was collected to perform the bisulfate conversion with a CpGenome™ DNA Modification kit (Millipore), according to the manufacturer’s instructions. PCR was used to amplify 287 bp from the binding region of the PTEN promoter region and the transcription factor Myc in the bisulfate-converted DNA. The amplification primer sequences were: 5′-TATTTATAAGGTGGAAGTTTTGAGG-3′ and 5′-ATAAAAAATAAACTCAACCCCACTC-3′. The PCR amplification conditions were 94°C for 3 min; 35 cycles of 94°C for 30 sec, 55°C for 30 sec and 72°C for 30 sec; and a final extension at 72°C for 7 min. The PCR products were cloned into a T-vector and transformed into *Escherichia coli* (*E. coli*) cells (DH5α). Subsequently, the *E. coli* were inoculated in Ampicillin^+^ (100 μg/ml) LB agar plates, incubated at 37°C for 12–16 h and then five independent clones were sequenced for the amplified fragment. The demethylation rate of the CpG pairs in the MG-63 cells treated with or without different concentrations of 5-Zac was calculated from the sequencing results.

#### Statistical analysis

SPSS software, version 16.0 for Windows (SPSS, Inc., Chicago, IL, USA) was used to store and analyze the data. The routine test of homogeneity of variance and the normality test were performed. Measurement and experimental data are expressed as the mean ± standard deviation. Multiple sets of data were compared using the F-test (one way analysis of variance). P<0.05 was considered to indicate a statistically significant difference.

## Results

### MG-63 cell growth is inhibited by 5-Zac

**Figure 1:**
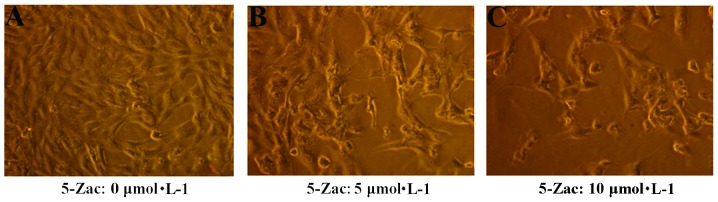
Morphology of the MG-63 cells by microscopy (magnification, 20×10) following treatment with different concentrations of 5-Zac after 72 h. 5-Zac, 5-azacytidine.

The morphology of the MG-63 cells was altered following the addition of 5-Zac (Fig. 1). The MG-63 cells in the standard RPMI-1640 complete medium were adherent and exhibited adequate growth (Fig. A), with the cells arranged similar to epithelia. The nuclear shape was round, the cell membrane was integrated, the cytoplasm presented a high degree of uniformity, and the cells possessed a high quality of refraction. Subsequently, 5-Zac (5 or 10 μmol/l) was added to the cells. After 72 h, cell proliferation was stagnated, numerous cells were crimpled, the vacuole was evident, the number of granules was increased in the cytoplasm, and high levels of impurity including cell fragments were identifiable (Fig. 1B and C).

### 5-Zac enhances PTEN mRNA expression levels in MG-63 cell

**Figure 2:**
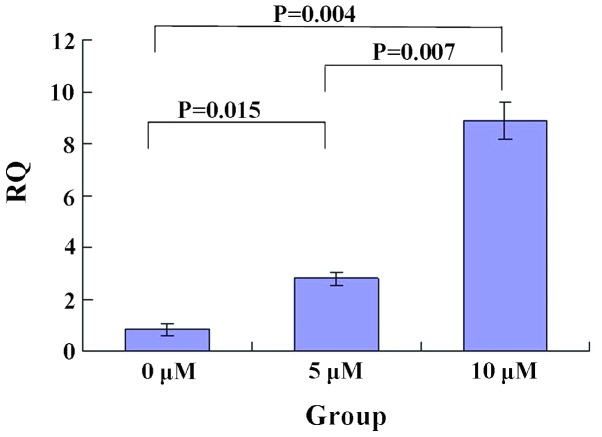
RT-PCR results of the levels PTEN mRNA in the MG-63 cells following treatment with different concentrations of 5-Zac. RQ, relative quantification; RT-PCR, reverse transcription-polymerase chain reaction; PTEN, phosphate and tension homolog; 5-Zac, 5-azacytidine.

**Figure 3:**
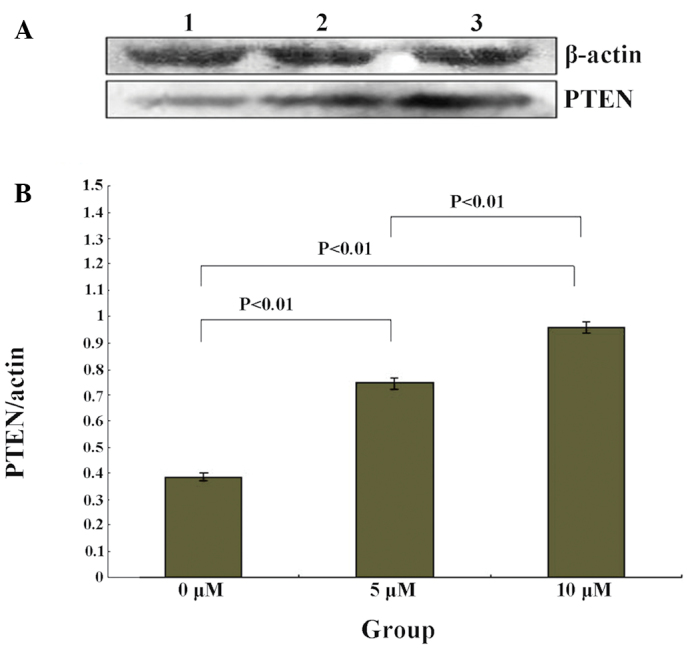
PTEN protein expression levels in the MG-63 cells following treatment with different concentrations of 5-Zac. (A) PTEN protein expression levels detected by western blotting. Bar 1: Group 0 μmol/l; bar 2: Group 5 μmol/l; and bar 3: Group 10 μmol/l. (B) Quantification of the PTEN protein expression levels. PTEN, phosphate and tension homolog; 5-Zac, 5-azacytidine.

The RT-PCR showed that 5-Zac increases the PTEN mRNA levels in a concentration-dependent manner (Figs. 2 and 3). The relative quantification (RQ) value of each group was 0.80±0.02 for the control group (0 μmol/l), 0.90±0.02 for the 5 μmol/l 5-Zac group, and 0.95±0.01 for the 10 μmol/l 5-Zac group. The statistical significance was calculated by comparing each combination of two groups: P<0.05 for the comparison between the 0 and the 5 and 10 μmol/l groups; and P=0.007 for the comparison between the 5 and 10 μmol/l groups.

### PTEN expression levels are upregulated by 5-Zac in MG-63 cells

The PTEN protein expression levels of the MG-63 cells were measured following treatment with different concentrations of 5-Zac. The results are shown in Fig. 3, which demonstrate that the PTEN protein expression levels increase with the increasing concentration of 5-Zac, and are concentration-dependent. The ratio between the PTEN protein and internal reference β-actin levels was 0.39±0.01 for the control group (0 μmol/l), 0.75±0.02 for the 5 μmol/l 5-Zac group, and 0.96±0.01 for the 10 μmol/l 5-Zac group. Also, significant statistical differences are shown among the three treatment groups, all P<0.01.

### 5-Zac induces apoptosis of MG-63 human osteosarcoma cells

**Figure 4:**
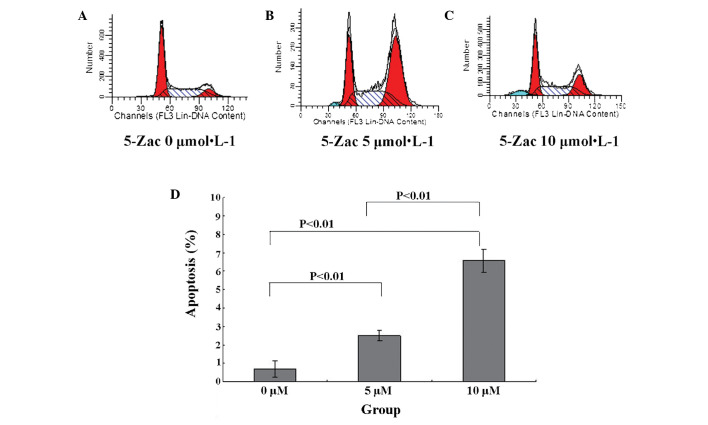
Apoptotic rate of the cells in each group treated with different concentrations of 5-Zac. (A–C) The apoptotic rate of the cells treated with 0, 5 and 10 μmol/l 5-Zac; and (D) comparison of the apoptotic rate for each 5-Zac concentration group. 5-Zac, 5-azacytidine.

The MG-63 cell apoptotic rate is 0.69±0.42% in the absence of 5-Zac. When 5-Zac is added to the cells and they are cultured for 72 h, the MG-63 cell apoptotic rate increases gradually, as Fig. 4 presents. It is observable that the cell apoptotic rate increases by 2.50±0.30% for the 5 μmol/l group and 6.59±0.62% for the 10 μmol/l group. Comparing the three groups, P<0.01 is obtained, and the differences are evidently statistically significant. Furthermore, the PTEN expression rate is higher in the cells treated with a higher concentration of 5-Zac.

### 5-Zac reduces the methylation of the PTEN promoter in MG-63 cells

**Figure 5:**
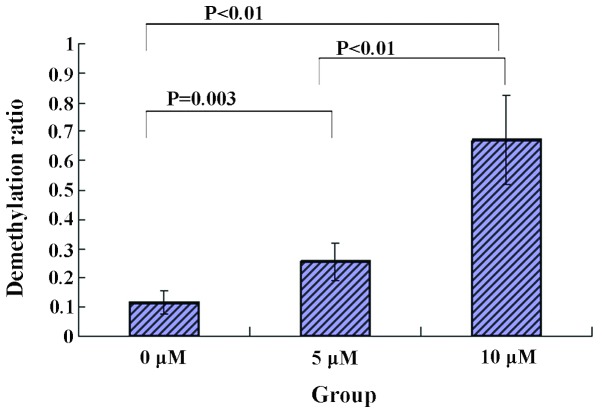
Average demethylation levels for each group treated with 0, 5 and 10 μmol/l.5-Zac. 5-Zac, 5-azacytidine.

The degree of methylation of 22 CpG points between −263 and 0 bp in the PTEN gene promoter was detected in each group of MG-63 cells following treatment with 5-Zac. Five clones from each group were selected for sequencing. The average methylation levels were produced following the sequencing. The results are shown in Fig. 5, where 1 represents full demethylation and 0, full methylation. It was found that the demethylation levels were largely increased for the CG points that bind to the transcription regulation factors Myc and Sp1 in the PTEN promoter; a large demethylation difference was evident for the 5 and 10 μmol/l groups by comparing with that of the 0 μmol/l group; the demethylation levels increase with the increasing concentration of 5-Zac; and the differences were evident by comparing the methylation among the three groups. Therefore, it is suggested that the methylation inhibitor 5-Zac affects the expression levels of PTEN in MG-63 cells possibly via the demethylation of the GC site that binds to the transcription factors Myc and Sp1 in the PTEN promoter.

## Discussion

Osteosarcoma is the most common type of primary malignant tumor in bone. The pathogenesis of osteosarcoma is complex and the precise molecular mechanisms have yet to be determined.

The present study cultured the MG-63 osteosarcoma cell line and treated the cells with different concentrations of the methylation inhibitor 5-Zac to detect the expression levels of the PTEN protein, the mRNA transcription levels of the PTEN gene, and the influence of the methylation status of the GC site that binds to the transcription factors Myc and Sp1 in the PTEN promoter.

It was demonstrated that the PTEN expression and mRNA transcription levels in the MG-63 cells gradually increased along with the increasing 5-Zac concentration, and the apoptotic rate of the MG-63 cells was also positively correlated to the 5-Zac concentration.

In a further experiment, it was revealed that following treatment with 5-Zac, the methylation status of the transcription factor binding fragment of the PTEN promoter had significantly changed.

These results suggest that following treatment with the methylation inhibitor 5-Zac, the PTEN expression and transcription levels in MG-63 osteosarcoma cells were significantly increased, and the number of apoptotic cells was increased. The gene transcription levels may be affected by methylation regulation. This study provides a novel perspective for future studies concerning the regulation mechanism of the PTEN gene, which is closely associated with osteosarcoma, and presents a novel theory that change in the methylation status of PTEN may be effective as a treatment for osteosarcoma.
